# Murine Models in Oral Research: A Narrative Review of Experimental Approaches and Cardiovascular Implications

**DOI:** 10.3390/biology14020127

**Published:** 2025-01-26

**Authors:** Asmaa Elhaieg, Ahmed Farag, Ahmed S. Mandour, Miki Hirose, Ahmed Elfadadny, Ryou Tanaka

**Affiliations:** 1 Veterinary Teaching Hospital, Tokyo University of Agriculture and Technology, Tokyo 183-8509, Japan; ahmedfarag9331@gmail.com (A.F.);; 2 Department of Surgery, Anesthesiology, and Radiology, Faculty of Veterinary Medicine, Zagazig University, Zagazig 44519, Egypt; 3 Department of Animal Medicine (Internal Medicine), Faculty of Veterinary Medicine, Suez Canal University, Ismailia 41522, Egypt; 4 Laboratory of Internal Medicine, Cooperative Division of Veterinary Sciences, Tokyo University of Agriculture and Technology, Fuchu 183-0054, Japan

**Keywords:** murine models, oral infections, periodontitis, cardiovascular disease

## Abstract

Oral research using murine models spans a broad spectrum of studies, including investigations into oral infections such as periodontitis and peri-implantitis, wound healing, periodontal responses to orthodontic treatment, and occlusal overload. This review aims to provide a comprehensive overview of murine models employed in oral research, with a particular focus on their relevance in studying systemic implications, including cardiovascular diseases (CVDs). The objectives of this review are twofold: first, to highlight the diversity of experimental methods utilized in murine oral research, such as ligature placement, bacterial inoculation, surgical interventions, and mechanical manipulations; second, to explore how these models enhance our understanding of oral–systemic interactions. The findings demonstrate that murine models have significantly contributed to uncovering how oral conditions influence systemic health. Models of oral infections reveal pathways linking systemic inflammation, endothelial dysfunction, and atherogenesis, while studies on wound healing and mechanical stress offer valuable insights into periodontal tissue responses and regeneration under various conditions. These diverse findings underscore the versatility of murine models in addressing key questions across oral health research. By replicating human disease mechanisms, murine models serve as powerful tools for investigating the interplay between oral health and systemic diseases, including cardiovascular dysfunction. The insights gained from these models guide the development of integrated therapeutic approaches aimed at mitigating systemic inflammation and promoting periodontal regeneration.

## Simple Summary

Murine models are invaluable for advancing oral research and understanding its systemic effects, including links to cardiovascular diseases (CVDs). This review highlights various experimental models, focusing on their methodologies and findings. Key insights reveal the role of systemic inflammation, bacterial dissemination, and tissue remodeling in connecting oral conditions to systemic health. These findings provide a foundation for developing therapeutic strategies to enhance both oral and cardiovascular health.

## 1. Introduction

Oral health is a critical component of overall systemic health, with conditions such as periodontal disease, periapical lesions, peri-implantitis, and orthodontic treatments leading to inflammation and plaque accumulation. These factors extend beyond localized tissue destruction, contributing to systemic health risks [1,2,3]. These chronic inflammatory diseases have been increasingly linked to systemic inflammatory cascades that influence distant organs, notably the cardiovascular system, contributing to conditions such as atherosclerosis, myocardial infarction, and endocarditis [1,4].

Cardiovascular diseases (CVDs) remain a leading cause of death, with a significant burden on healthcare systems [5,6]. Atherosclerotic cardiovascular diseases (ACVD), including coronary heart disease and stroke, are particularly prevalent. Studies suggest a link between oral infections, particularly periodontitis, and ACVD, primarily through chronic inflammation and elevated inflammatory markers. These factors, alongside molecular mimicry and immune responses, contribute to the relationship between oral health and cardiovascular disease [7]. Epidemiological studies, such as one using data from the Korea National Health and Nutrition Examination Survey, have found that individuals with cardiovascular risk factors are more likely to have periodontal disease. The study emphasizes the need for regular oral health evaluations and education in at-risk populations to support primary and secondary prevention of cardiovascular diseases [8].

The use of murine models has proven instrumental in exploring the mechanistic links between oral infections and cardiovascular impairments. These models enable researchers to control experimental variables rigorously and to simulate the pathophysiological conditions of human cardiovascular diseases [9,10,11]. Popular models, such as the periodontal ligature model, periapical lesion induction, and peri-implantitis model, are widely used for this purpose. For instance, pathogens commonly associated with periodontal disease, such as *Porphyromonas gingivalis* and *Aggregatibacter actinomycetemcomitans*, have been shown to stimulate systemic inflammatory pathways, increasing cytokine levels and induce vascular changes, which support the hypothesis that oral infections can exacerbate cardiovascular diseases [12,13].

This review hypothesizes that murine models are essential for unraveling the multifaceted links between oral conditions and systemic health, particularly cardiovascular diseases (CVDs). By examining key inflammatory mediators, such as interleukin-6 (IL-6), tumor necrosis factor-alpha (TNF-α), and C-reactive protein (CRP), as well as mechanisms like bacterial translocation and oxidative stress, these models offer unparalleled insights into how oral health influences cardiovascular function [14,15]. This manuscript offers a narrative review that summarizes the diversity of murine models used in oral research. It synthesizes findings on their methodologies, key outcomes, and translational relevance. The review aims to guide future research by highlighting the importance of murine models in exploring the relationship between oral and systemic health and identifying opportunities to optimize therapeutic strategies.

In preparing this review, a comprehensive literature search was conducted using scientific databases such as Google Scholar, Scopus, and Web of Science. Key search terms included “murine models of oral infections”, “oral disease and cardiovascular health”, “periodontitis animal models”, and “systemic inflammation from oral pathogens”. Articles were selected based on relevance to the focus of murine models simulating oral diseases and their cardiovascular implications. Studies from peer-reviewed journals published in the past two decades were prioritized to ensure current and robust data.

## 2. Experimental Models

### 2.1. Surgical Models

#### 2.1.1. Periodontal Model

To induce periodontal infection, 4/0 non-resorbable sterile silk ligatures were placed in an “8” configuration around the inferior frontal teeth of adult Wistar rats, leading to gingival irritation, plaque accumulation, and periodontal disease over 14 days [16]. In another variation, 5-0 silk ligatures were carefully positioned around the left maxillary second molar in female C57BL/6 mice, with the contralateral molar serving as an internal control to minimize periodontal tissue damage [17]. In a different approach, a 0.3 mm elastic band was inserted between the first and second molars on the right upper jaw of mice (OTM-side) after one week on either a low, normal, or high-salt diet. This setup induced mechanical stress and periodontal inflammation [18]. Additionally, in the C57BL/6J mouse model, periodontitis was induced through two methods: a ligature model and an oral gavage model. Mice in the ligature group received molar ligatures to stimulate inflammation and bone loss, while those in gavage groups were inoculated orally with *Porphyromonas gingivalis* alone or in combination with *Fusobacterium nucleatum* at two-day intervals. After 45 and 60 days, assessments were conducted using stereometry, microcomputed tomography, histometry, and qPCR to confirm bacterial colonization. Only the ligature model resulted in substantial inflammation and bone resorption [19].

#### 2.1.2. Ligature-Induced BRONJ Model

In this model designed to simulate Bisphosphonate-Related Osteonecrosis of the Jaw (BRONJ), a ligature was placed around the right maxillary first molar of rats to induce periodontal disease. Concurrently, zoledronic acid, a potent bisphosphonate, was administered, resulting in alveolar bone loss and osteonecrosis at the ligation site. Microcomputed tomography and histologic analysis confirmed necrotic bone features comparable to BRONJ in humans [20].

#### 2.1.3. Zoledronate-Induced Osteonecrosis of the Jaw (ONJ) Model

This model investigated the role of γδ T cells in ONJ development. Female C57Bl/6J mice received an intravenous dose of zoledronate (540 μg/kg), followed by extraction of the maxillary left first molar after one week. In wild-type mice, wound healing was delayed, resulting in open wounds and epithelial hyperplasia, with γδ T cells detected in the extraction sites. In contrast, Tcrd−/− mice (γδ T cell-deficient) treated with zoledronate exhibited better wound healing, suggesting that γδ T cells modulate oral mucosal responses but are not directly involved in osteonecrosis mechanisms [21].

#### 2.1.4. Tooth Movement Model

In this orthodontic model, 24 male Wistar rats received a constant force of 0.25 N applied to the left first upper molar and upper incisors using a modified NiTi coil spring for up to four weeks. Rats were sedated with xylazine and ketamine, positioned with a transpalatinal bar to immobilize the upper jaw, and their mouths disinfected with chlorhexidine. The coil spring was attached and secured between the incisors, with weekly trimming to accommodate natural eruption [22]. In a variant, 68 male Wistar rats under anesthesia had a miniscrew inserted behind the upper incisors, with a NiTi coil spring applying a 50 g force to the left upper first molars for controlled tooth movement [23].

#### 2.1.5. Periapical Lesion Induction

Periapical lesions were induced in 60-day-old female Sprague-Dawley rats, anesthetized, and positioned on a jaw retraction board. Using a low-speed handpiece with a #1/2 round bur, pulpal exposures were created in the first and second molars in both maxillary and mandibular arches, avoiding the furcae. The exposed teeth were left open to the oral cavity, promoting infection development [24].

#### 2.1.6. Unilateral Anterior Crossbite Model

To induce a crossbite, 6-week-old female C57BL/6J mice were anesthetized, and metal tubes were bonded to the left maxillary and mandibular incisors using zinc phosphate cement. Maxillary tubes measured 1.5 mm in length, while mandibular tubes were curved into a 135° plate for crossbite formation. Control mice underwent the same procedures without tube placement [25].

#### 2.1.7. Dental Occlusion Model

For hyperocclusive conditions, Swiss–Webster mice were anesthetized, and their right maxillary molars were acid-etched with 30% phosphoric acid. Stainless steel wire segments were bonded to the molars using a resin adhesive to create an occlusive state [26]. A similar method used a custom dental platform to keep the mice’s mouths open while stainless steel wire was applied and secured with flowable composite, simulating occlusal stress [27].

#### 2.1.8. Medial and Distal Molar Displacement Model

In this occlusion-disordered model, 1 mm elastic bands were inserted between the first and second molars to displace the first molars medially, followed by resin replacement. After four weeks, the third molars were displaced distally. Animals were sacrificed at 8 or 12 weeks for analysis [28].

#### 2.1.9. Mandibular Incisor Trimming Model

To examine occlusal effects, CD-1 female mice underwent periodic mandibular incisor trimming, removing approximately 1 mm of tooth structure every other day. Mice were divided based on dietary and surgical treatments, and experimental durations ranged from 2 to 6 weeks [29].

#### 2.1.10. Forced Mouth Opening Model

In this model, custom-made stainless steel springs applied a force of either 0.25 N or 0.50 N to ensure a maximal mouth opening of 10 mm, with recalibrations performed daily. The procedure continued for five consecutive days, after which the mice were sacrificed for analysis [30].

#### 2.1.11. Implantation Model

In this model, 21 male Sprague-Dawley rats underwent maxillary implantation. After anesthesia and immobilization, access to the maxilla was achieved using retractors. The left maxillary second molar was extracted, and a socket was prepared by removing the interradicular bone with a dental bur. A chlorhexidine-disinfected titanium implant was then inserted, positioned to contact both hard and soft palatal tissues, and secured with acrylic resin. To prevent occlusal interference, the opposing mandibular teeth were extracted. Rats were housed individually post-surgery and monitored with access to standard chow and water. Resuscitation, when needed, involved oxygen and cardiac compression [31]. Another study used adult male CD1 mice (3–5 months old) for a similar implantation procedure. Following anesthesia with ketamine and xylazine, a sulcular incision was made from the first molar to the incisor, and a full-thickness flap was elevated. A pilot hole was drilled into the alveolar crest using 0.3 mm and 0.45 mm drill bits. A titanium implant (0.6 mm in diameter, 2 mm long) was placed, leaving part exposed. Control animals had sites rinsed and sutured without implants. Post-surgery, mice received buprenorphine and were sacrificed at specific intervals for analysis [32]. In a model using 4-week-old Wistar rats, the right maxillary first molar was extracted under chloral hydrate anesthesia. After four weeks, a bone cavity was created with a K-Reamer, and custom titanium implants (3.5 mm long, 2 mm diameter) were inserted. Rats were divided into loading and non-loading groups, with an abutment attached in the loading group four weeks post-implantation. All rats were euthanized eight weeks after implantation for further analysis [33].

#### 2.1.12. Peri-Implantitis Model

In this model, 4-week-old C57BL/6J male mice underwent extraction of the left maxillary first, second, and third molars under anesthesia, followed by an eight-week healing period. Titanium implants were placed into the healed sockets, and a four-week osseointegration period followed. In the experimental group, 6-0 silk ligatures were tied around the implants to induce peri-implantitis, while control animals received no ligatures. Mice were sacrificed 12 weeks post-ligation, and the maxillae were fixed in paraformaldehyde for analysis [34].

#### 2.1.13. Diabetic Peri-Implantitis Model

This model aimed to simulate peri-implantitis under diabetic conditions (T2DM-PI) in Sprague-Dawley rats. Following extraction of the maxillary first molars, customized cone-shaped titanium implants were installed in the sockets. Type 2 diabetes was induced through a high-fat diet and streptozotocin injections. To stimulate peri-implantitis, lipopolysaccharide (LPS) was injected into the implant sulcus, resulting in significant inflammatory cell infiltration, increased cytokine expression, and enhanced matrix metalloproteinase (MMP) activity, which promoted collagen breakdown and bone resorption [35].

#### 2.1.14. Oral Floor Tumor Model

In this tumor induction model, female BALB/c-NU mice received anesthesia with Rompun/Xylazine and Ketanest. Tumor cells (2 × 10^6^ or 8 × 10^5^) suspended in Matrigel/HBSS were injected trans-cervically into the floor of the mouth. Once tumors reached 0.8 cm, they were surgically excised. Using a binocular microscope, a horizontal skin incision was made to expose and excise the tumor along with a safety margin. Hemostasis was achieved with micro-bipolar forceps, and resection was confirmed using fluorescent and photon imaging. Nylon sutures were used to close the wound, and buprenorphine was provided for post-operative pain management [36].

#### 2.1.15. Simulated Alveolar Cleft Model

An anesthetized rat model (ketamine and xylazine) was positioned dorsally to create an anterior maxillary alveolar cleft by making a sagittal incision along the mid-palatal suture. A mucosal flap was lifted, and a 3.3 mm bone defect was drilled. Treatments included no graft, bone graft (bHA), bHA with undifferentiated mesenchymal stem cells (MSCs), or bHA with osteogenically differentiated MSCs. The flap was sutured post-treatment, and animals received antibiotics and pain relief, soft diets, and regular monitoring. Bone formation was later assessed using fluorochrome dye injections prior to euthanasia [37].

#### 2.1.16. Orthodontic Tooth Movement (OTM) Model

In this model, 10-week-old C57BL6/J mice were anesthetized and positioned with a custom mouth opener for full intraoral access. The right first maxillary molar and incisors were cleaned, etched, and bonded with a NiTi open-coil spring. The distal end of the spring was attached to the first molar and the anterior end to a tension gauge. Different orthodontic forces (0.10 N, 0.25 N, 0.35 N, and 0.50 N) were applied, with the upper incisors used as an anchorage control. Mice were sacrificed at designated time points for molecular and histopathological assessments [38].

#### 2.1.17. Root Resorption Model

To induce root resorption, rats underwent the application of a 50 g force to the left first molar using a NiTi closed-coil spring, secured with orthodontic ligature wire under anesthesia. The coil spring was attached to the ipsilateral incisor, reinforced with light-polymerized flowable composite to prevent detachment, maintaining mesial pull on the molar for 21 days to promote resorption [39].

#### 2.1.18. Tooth Extraction Model

This model involved the extraction of the upper right incisor from anesthetized mice (ketamine and xylazine) under 25× magnification with a stereomicroscope. Using a dental probe, the tooth was gently luxated and removed with clinical tweezers. Integrity checks excluded animals with fractured teeth. Mice were sacrificed at specific time points (0 h, 7, 14, and 21 days post-extraction) for collection and analysis of the maxillae using micro-CT, histological, and molecular methods [40].

#### 2.1.19. PCL Biomembrane Implantation in Maxillary Defects Models

Under general anesthesia, a maxillary bone defect was created in the diastemal area of mice using a 500 μm dental bur following a gingival incision. Three types of scaffolds—bifunctionalized BMP-2/Ibuprofen, functionalized Ibuprofen, or functionalized BMP-2—were implanted into the lesion on one side, while the other side served as a control with either no scaffold or a nonfunctionalized membrane. The mucosa was closed using biological glue (3M Vetbond™ Tissue Adhesive, Fisher Scientific, Illkirch, France) to secure the site [41].

#### 2.1.20. Oral Mucosa Wound Healing Model

This model assessed the effects of Enamel Matrix Protein Derivative (EMD) on oral mucosal wound healing in Sprague-Dawley rats. Bilateral incisions were made in the anterior edentulous maxilla, with EMD injected into the soft tissue on one side and a vehicle control administered on the contralateral side. After 5 and 9 days, histological and immunohistochemical analyses were conducted, showing that EMD treatment significantly increased blood vessel formation and collagen content in connective tissue. EMD also enhanced the expression of key growth factors and inflammatory cytokines, promoting vascularization and collagen synthesis without affecting the epithelial gap [42].

#### 2.1.21. FRICTION Model

The FRICTION Model (Foramen Rotundum Inflammatory Constriction of the Trigeminal Infraorbital Nerve) was developed to study chronic neuropathic pain resulting from trigeminal nerve injury. In this model, a chromic gut suture is inserted orally to irritate the V2 branch of the trigeminal nerve, inducing pain without visible injury. This method enables long-term evaluation (up to 100 days) of mechanical hypersensitivity and behavioral changes, including anxiety and depression associated with chronic pain. The model supports blinded studies and is effective for testing non-opioid pain therapies. Validation in both BALB/c and C57BL/6 mouse strains confirms the model’s reproducibility [43].

#### 2.1.22. Tunable Mechanical Overload Model

This model for temporomandibular joint osteoarthritis (TMJ-OA) in female Holtzman rats uses tunable mechanical overload to induce orofacial pain. Two magnitudes of jaw-opening force (2 N and 3.5 N) were applied for one hour daily over seven days, followed by a rest period. Orofacial sensitivity and joint degeneration were assessed, with the 3.5 N force significantly decreasing head-withdrawal thresholds and resulting in cartilage degradation typical of TMJ-OA. This group also showed upregulation of inflammatory markers, such as MMP-13, HIF-1α, and TNF-α, while the 2 N group did not display persistent pain or the same inflammatory response. This model differentiates between acute and chronic TMJ-OA pain mechanisms, facilitating the investigation of potential treatments [44].

### 2.2. Drug/Chemically Induced Rodent Models

#### 2.2.1. 4NQO-Induced Oral Mucosal Carcinogenesis Models

To induce oral mucosal carcinogenesis, Sprague-Dawley rats were exposed to 4-nitroquinoline-1-oxide (4NQO) by applying the carcinogen to the palatal and tongue mucosa three times per week for four weeks. Following this initial exposure, the rats were either treated with a phorbol ester as a promoter, infected with *Candida albicans*, or left untreated. At 34 and 52 weeks, the rats were euthanized, and histological analysis of their tongues and palates was performed to assess neoplastic changes [45]. Similarly, male C57BL/6JNarl mice were given combinations of 4NQO (100 or 200 μg/mL) and arecoline hydrobromide (250 or 500 μg/mL) dissolved in drinking water over eight weeks, with oral lesions monitored bi-weekly. Mice were sacrificed at 8 or 28 weeks, and histological examination and survival rates were assessed [46]. In a related study, CBA and C57Bl/6 mice were treated with 4-nitroquinoline 1-oxide (4-NQO) either via tongue painting or in their drinking water. The treatment led to significant tumor development in the oral cavity, manifesting as dysplasias, papillomas, and invasive squamous cell carcinomas. Immunohistochemical analysis revealed altered expression of keratin markers, increased cell proliferation, reduced p16 expression, and elevated epidermal growth factor receptor levels. Similar carcinogenic effects were observed in the esophagus, while other organs remained unaffected, making this model suitable for investigating oral and esophageal cancer development and testing therapeutic intervention [47].

#### 2.2.2. Osteonecrosis of the Jaw (ONJ) Model

In this model, high doses of zoledronic acid were administered to mice to simulate the effects of bisphosphonates. To induce osteonecrosis, the pulpal exposure of mandibular molars was performed to create periapical disease. After eight weeks, the animals underwent radiographic and histological analyses to evaluate ONJ characteristics [48].

#### 2.2.3. Bisphosphonate-Related and Denosumab-Related Osteonecrosis of the Jaw (BRONJ and DRONJ) Models

In the BRONJ model, female C57BL/6 mice were injected intravenously with 125 μg/kg of zoledronic acid (ZOL) or saline biweekly for four weeks. In the DRONJ model, mice were injected biweekly with 250 μg of an anti-mouse RANKL monoclonal antibody or control IgG. One week after the initial injection, maxillary first molars were extracted atraumatically. Three weeks post-extraction, animals were euthanized, and maxillae, femurs, and blood were harvested. Samples underwent micro-computed tomography, decalcification, and histological processing [49].

#### 2.2.4. Oral Ulcers Model

In this model, Wistar rats were assigned to groups with intact salivary glands or reduced salivary secretion following sialoadenectomy, which involved the removal of the submandibular and sublingual glands. Two weeks post-surgery, rats were anesthetized, and 100% acetic acid was applied to the gingival or lingual mucosa to induce ulcers. Rats then received intraperitoneal injections of either saline or ghrelin (4, 8, or 16 nmol/kg/dose) twice daily for six days to evaluate ulcer healing [50]. Another variant used young adult male Wistar rats, anesthetized and subjected to gingival ulcers induced with a formocresol-soaked cotton swab applied to the gingiva for 60 s. Ulcers were evaluated 48 h later [51].

#### 2.2.5. Dentin Protein Immunization Model

In this model, six NIH Swiss female mice were immunized intraperitoneally with 100 µg of mouse dentin protein in Complete Freund’s Adjuvant (CFA), while control mice received CFA alone. Four weeks later, all mice received an Incomplete Freund’s Adjuvant (IFA) booster, with or without dentin protein, followed by two additional weekly boosters with mouse dentin and later Sprague-Dawley rat dentin. Weekly blood samples were collected to monitor antibody production. After the final booster, incisors were exposed to cryoprobe treatment, and mice were sacrificed 10 days later for analysis [52].

#### 2.2.6. Induced Buccal Mucosa Lesion Model

Adult female BALB/c mice were divided into control and test groups for 300, 350, 450, or 600-day treatment durations. The test group received 35 µL of aqueous areca nut extract (265 g/L in saline) applied to the buccal mucosa twice daily, while control animals received saline. Water was withheld for two hours post-application, with treatments conducted six days per week. Mice were euthanized at the end of the treatment period, and buccal mucosa tissues were collected, fixed, and prepared for analysis [53].

#### 2.2.7. Deproteinized Bovine Bone and Bioactive Glass-Induced Guided Tissue Regeneration (GTR) Model

This model was developed to evaluate the long-term effects of deproteinized bovine bone and bioactive glass on bone formation in rat mandibles. Hemispherical Teflon capsules were filled with either deproteinized bovine bone (Bio-Oss^®^) or bioactive glass (Biogran^®^) and placed on the mandibular ramus to establish a defined bone defect. After one year, histological analysis revealed that bone formation was limited in the test groups, with 23% new bone formation for Bio-Oss^®^ and 12.6% for Biogran^®^. By contrast, control animals with empty capsules showed 88.2% new bone formation. This model demonstrates the inhibitory effects of these grafting materials on bone regeneration within the GTR framework [54].

#### 2.2.8. Oral Submucous Fibrosis Model

This rat model of oral submucous fibrosis was created using bleomycin injections to replicate human OSF. In a randomized trial, test rats received daily injections of bleomycin diluted in phosphate-buffered saline, while control rats received saline alone. Injections were administered into the buccal mucosa for 2, 4, 6, or 8 weeks. The protocol involved evaluating histopathological characteristics, quantifying myofibroblast levels, and analyzing collagen types I and III, along with transforming growth factor–beta 1 and interferon-γ levels. Significant alterations in the buccal mucosa, including increased collagen deposition and ultrastructural changes, were observed in the test rats, closely resembling the fibrotic conditions found in human OSF [55].

### 2.3. Genetically Induced Rodent Models

#### 2.3.1. Induced Oral Cancer Model

This mouse model for oral cancer utilizes mice with inducible K-rasG12D mutations in stratified epithelial tissues. Mice carrying the Lox-stop-Lox (LSL)-K-rasG12D allele were crossed with K14.CrePR1 or K5. CrePR1* mice, which express Cre recombinase under keratin promoters. To activate the K-rasG12D allele, RU486 was administered to excise the stop cassette, resulting in oncogenic K-ras expression in oral tissues. This genetic modification led to the formation of benign squamous papillomas, closely resembling early stages of human oral cancer. Tissue samples were collected, fixed, embedded in paraffin, sectioned, and stained for histological examination [56].

#### 2.3.2. Induced Cyclin D1/p53 Tumorigenesis Model

Transgenic mice expressing human cyclin D1 were generated by microinjecting cyclin D1 cDNA into single-cell embryos from superovulated D3/C57BL/6 females, followed by reimplantation into foster females. Founder mice were identified using Southern blot and PCR analysis. The transgenic mice were then backcrossed with C57BL/6 and p53 heterozygous mice to enhance tumorigenesis, yielding various cyclin D1 and p53 genotype combinations. Additionally, a subset of wild-type and L2D1+/p53+/− mice were treated with 400 ppm sulindac in drinking water for three months to evaluate its chemopreventive potential. Mice were sacrificed one month post-treatment for analysis [57].

#### 2.3.3. TGF-β3 Knockout Model for Palatal Fusion Studies

The TGF-β3 knockout model was generated by breeding heterozygous (TGF-β3+/−) mice to produce homozygous knockout (TGF-β3−/−), heterozygous (TGF-β3+/−), and wild-type (TGF-β3+/+) embryos. Pregnant females were sacrificed on embryonic days 12.5–16.5 to retrieve embryos, which were then genotyped using PCR with specific primers for the TGF-β3 gene. Palatal shelves were dissected from embryos at E13.5 or E14.5 and cultured in vitro using the Trowell organ culture method. Various TGF-β isoforms, including rh TGF-β1, p TGF-β2, and rh TGF-β3, as well as other growth factors, were added to the culture medium at different concentrations and time points to investigate their effects on palatal shelf fusion [58].

### 2.4. Pathogen-Induced Rodent Models

#### 2.4.1. Infection-Induced Periapical Lesion Model

In this model, periapical lesions were induced in genetically engineered IL-4−/− and IL-10−/− mice, along with wild-type controls, by infecting exposed molar pulps. Mice were anesthetized using ketamine and xylazine, and the pulps of all four first molars were exposed via a dental bur under a surgical microscope. A mixture of four endodontic pathogens—*Prevotella intermedia*, *Fusobacterium nucleatum*, *Peptostreptococcus micros*, and *Streptococcus intermedius*—was prepared anaerobically and inoculated directly into the exposed pulps. Mice with unexposed pulp served as negative controls [59].

#### 2.4.2. Polymicrobial Periodontal Infection Model

This model investigated the effects of polymicrobial infection with *Porphyromonas gingivalis*, *Treponema denticola*, and *Tannerella forsythia* on periodontal inflammation and alveolar bone resorption in rats. Rats were infected with either monomicrobial or polymicrobial combinations, with some groups also receiving *Fusobacterium nucleatum*. PCR analyses confirmed the coexistence and interaction of these pathogens in the oral environment. The results showed significantly greater bone resorption in polymicrobial infections compared to monomicrobial infections or controls, indicating a synergistic effect in promoting chronic inflammation and bone loss [60].

#### 2.4.3. Peri-Implant Mucositis and Peri-Implantitis Model

Four-week-old C57BL/6J male mice underwent maxillary molar extraction under anesthesia, with an eight-week healing period followed by custom titanium implant placement in the edentulous ridge. Oral antibiotics were administered post-implantation for four weeks. To induce peri-implant mucositis and peri-implantitis, 2 µL of *Porphyromonas gingivalis* LPS (10 mg/mL) was injected into the peri-implant mucosa twice weekly for six weeks. Control groups received either no injections or PBS injections. Mice were euthanized post-treatment, and maxillae were fixed for analysis [61].

#### 2.4.4. *Candida albicans* Colonization Model

To study *Candida albicans* colonization, 48 rats were inoculated with *C. albicans* and divided into two groups: one received metronidazole treatment, while the other received plain water as a control. Colonization levels were assessed on days 1, 2, 5, and 7 post-inoculation, with follow-up collections at 15-day intervals up to 18 total assessments. Rats were sacrificed at 7, 15, or 30 days, and a histological examination of tongue samples was conducted [62].

#### 2.4.5. Pathogen-Induced Periodontal Disease Model

In this model, 108 mice were used to study periodontal disease. Mice were anesthetized, and a nylon thread ligature was placed around the first maxillary molar to induce periodontal inflammation. *Porphyromonas gingivalis* (Pg) and *Fusobacterium nucleatum* (Fn) were cultured anaerobically, and mice were infected with these bacteria in monomicrobial or polymicrobial configurations. In addition, heat-killed Pg was injected into the palatal mucosa in some groups. Negative controls received phosphate-buffered saline (PBS) injections. In another protocol, SPF mice pre-treated with antibiotics were infected with live Pg (10^9^ CFU) in PBS with 2% carboxymethylcellulose, administered both orally and intragastrically. Control mice received the same antibiotic treatment without bacterial infection, and all were euthanized 47 days post-infection for analysis [63,64].

#### 2.4.6. Inflammatory Bone Loss Model

This model used Mkp-1+/+ and Mkp-1−/− mice to study inflammatory bone loss. Alveolar bone loss was induced by injecting 20 µg of *Aggregatibacter actinomycetemcomitans* LPS in 2 µL PBS into the palatal interproximal gingiva between the first and second molars on the left side. PBS was injected into the corresponding right side as a control. Injections were administered three times a week for four weeks, followed by euthanasia and fixation of maxillae in 10% buffered formalin for analysis [65].

#### 2.4.7. Pathogen-Induced BRON Model in Rats

This model was designed to study Bisphosphonate-Related Osteonecrosis (BRON) in both the jaw and long bones of Wistar rats. Rats received weekly subcutaneous injections of zoledronic acid (Group Z) or saline (Group C) for four weeks. Following this treatment, *Aggregatibacter actinomycetemcomitans* (Aa), complete Freund’s adjuvant (CFA), or saline was injected into the bone marrow of the mandibles and femurs. After four weeks, histological examination revealed that zoledronic acid combined with Aa or CFA led to extensive osteonecrosis in both mandible and femur bones, indicating that inflammatory stimuli can enhance BRON development across different bone types [66].

#### 2.4.8. Bacterial Lavage-Induced Peri-Implantitis Model in Rats

This model was developed to simulate peri-implantitis in Sprague-Dawley rats through mixed bacterial infection. Two titanium implants were placed in each rat’s maxilla, followed by a 3-week healing period. Rats were then divided into three groups: the antibiotic group, receiving antibiotic oral lavage; the bacteria group, receiving oral lavage with *Streptococcus oralis* and *Aggregatibacter actinomycetemcomitans*; and the untreated group, which received no additional treatment. After three months, microscopic and histological analyses indicated that the bacteria group experienced significantly greater bone loss and inflammation around the implant sites, mimicking the inflammatory and bone-loss characteristics of human peri-implantitis [67].

#### 2.4.9. Dental Implant Biofilm Formation Model

**Figure 1:**
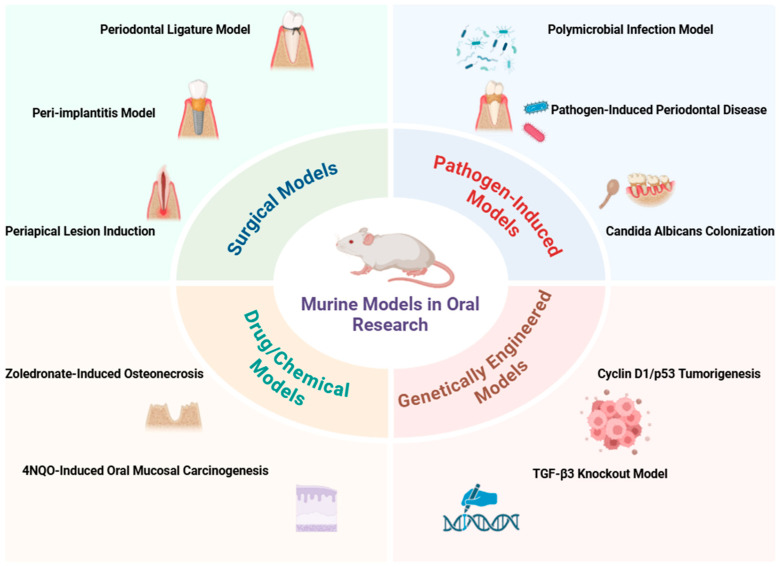
Overview of experimental murine models used in oral research.

This study established a rodent model using female Sprague-Dawley rats to investigate biofilm formation and peri-implant health on custom-made dental implants. The model involved a three-step implantation procedure, after which the rats were divided into two groups: Group A received continuous antibiotic treatment for seven weeks, while Group B was repeatedly inoculated with human-derived strains of *Streptococcus oralis*, *Fusobacterium nucleatum*, and *Porphyromonas gingivalis* over six weeks. This innovative approach allows for reliable biofilm quantification and assessment of peri-implant mucositis, providing a valuable platform for evaluating new implant materials and modifications aimed at reducing bacterial colonization [68]. Figure 1 and Table 1 summarize the various experimental murine models used for oral research, including their methodologies, species used, and key findings.

## 3. Cardiac Implications of Oral Infections

### 3.1. Mechanisms Linking Oral Infection to Cardiac Dysfunction

#### 3.1.1. Bacterial Dissemination and Inflammatory Pathways

A growing body of evidence suggests that oral infections, particularly periodontitis, are closely associated with systemic inflammation, which can severely impact cardiovascular health [69]. Periodontitis, marked by progressive destruction of the alveolar bone, has been linked to cardiac dysfunction via inflammation-driven mechanisms. In animal studies, ligature-induced periodontitis in rats has demonstrated declines in cardiac function, including decreased ejection fraction and impaired myocardial strain rates. These cardiac alterations, accompanied by histopathological evidence of myocardial damage, indicate that systemic inflammatory responses from oral infections can contribute to cardiovascular deterioration [10].

Pathogens such as *Porphyromonas gingivalis* activate systemic inflammatory pathways, significantly elevating pro-inflammatory cytokines like interleukin-6 (IL-6). This response promotes atherosclerosis by fostering vascular inflammation and endothelial dysfunction. The presence of *P. gingivalis* DNA in aortic tissue further suggests the direct involvement of periodontal pathogens in vascular pathology. Enhanced expression of vascular cell adhesion molecules and tissue factors in infected mice demonstrates increased vascular activation, linking periodontal infections to cardiovascular complications [70]. Similarly, *Aggregatibacter actinomycetemcomitans* induces a robust systemic inflammatory response, with elevated levels of IL-6, IL-8, and TNF-α and its bacterial DNA detected in the blood, heart, and spleen. The expression of vascular adhesion molecules like ICAM-1 and E-selectin, along with Toll-like receptors (TLR2 and TLR4) in the aorta, indicates heightened vascular inflammation and activation associated with this pathogen [71].

Inflammatory dental diseases can also elevate plasma fibrinogen and white blood cell counts, promoting a hypercoagulable state that impairs endothelial function. These changes are central to myocardial infarction pathogenesis [72]. Additionally, *P. gingivalis*-induced lipopolysaccharides (LPS) can stimulate hyperlipidemia and activate polymorphonuclear leukocytes (PMNs), which release pro-inflammatory cytokines like IL-1β, contributing to endothelial dysfunction, atherosclerosis, and increased coronary artery disease risk [73]. Other oral infections, including peri-implantitis, also play a role in cardiovascular disturbances through elevated serum levels of triglycerides and uric acid, promoting systemic cardiovascular risk [74,75].

Chronic apical periodontitis (CAP) induced by *P. gingivalis* triggers systemic inflammation and plays a crucial role in cardiovascular health. Periodontal pathogens stimulate pro-inflammatory cytokine release, resulting in vascular inflammation and endothelial dysfunction, which enhances atherosclerosis through plaque formation. CAP also influences gut microbiota, further exacerbating systemic inflammation and cardiovascular risks [76]. Experimentally induced apical periodontitis (AP) has been linked to oxidative stress, particularly in hypertensive rats, with elevated superoxide anion levels leading to myocardial damage and impaired cardiac dynamics. Reduced antioxidant enzyme activity in AP further exacerbates oxidative damage in cardiac tissues. This inflammatory burden likely explains the observed correlation between the severity of oral infection and the extent of cardiovascular impairment [77,78]. AP is also associated with endothelial dysfunction (ED), marked by elevated endothelin-1 (ET-1) and intercellular adhesion molecule-1 (ICAM-1), which disrupts vascular integrity and fosters atherogenesis [79].

Experimental bacteremia with *P. gingivalis* has also been shown to trigger myocarditis and myocardial infarction by stimulating pro-inflammatory cytokines. Elevated levels of IL-1β, IL-6, IL-17A, IL-18, TNF-α, and IFN-γ in heart tissue indicate a robust inflammatory response, where IL-17A is crucial for mediating cardiac inflammation. Neutrophil and monocyte infiltration in the hearts of infected animals further underscores the impact of *P. gingivalis*-induced inflammation in promoting cardiac dysfunction [80]. Additionally, *Treponema denticola*, a spirochete associated with periodontitis, contributes to systemic inflammation, shown by elevated IgG antibodies, which promote vascular inflammation and endothelial dysfunction. This pathogen also elevates oxidized low-density lipoprotein (LDL) and reduces nitric oxide (NO) levels, worsening vascular health and advancing atherosclerosis [81].

Recent studies also emphasize the systemic implications of oral infections in cardiovascular health. For example, a study investigated the protective effects of GV1001, an anticancer vaccine with anti-inflammatory and antioxidant properties, in mitigating the impact of *P. gingivalis*-induced periodontal disease on atherosclerosis and Alzheimer’s disease (AD) in Apolipoprotein E (ApoE)-deficient mice. The findings suggest that GV1001 effectively suppressed the development of periodontal disease, atherosclerosis, and AD-like conditions by modulating local and systemic inflammation. The vaccine worked by reducing the accumulation of *P. gingivalis* DNA aggregates, lipopolysaccharides (LPS), and gingipains in the gingival tissue, arterial wall, and brain. Notably, GV1001 inhibited vascular inflammation, lipid deposition in the arterial wall, and endothelial-to-mesenchymal cell transition (EndMT), which are key processes in atherosclerosis development. Additionally, GV1001 suppressed the accumulation of AD biomarkers in the brains of infected mice, presenting it as a promising preventive agent in oral pathogen-associated systemic diseases [82]. Moreover, another study explored how the disruption of circadian rhythms exacerbates *P. gingivalis*-induced atherosclerosis. Using various mouse models, including Bmal1−/− (brain and muscle Arnt-like protein 1) and ApoE−/− mice, the study demonstrated that *P. gingivalis* infection accelerated atherosclerosis progression by triggering oxidative stress and inflammatory responses in the arteries. The mechanistic dissection revealed that *P. gingivalis* infection activates the TLRs-NF-κB signaling axis, which subsequently recruits DNMT-1 to methylate the BMAL1 promoter and suppress its transcription. The downregulation of BMAL1 allowed CLOCK to phosphorylate p65, further enhancing NF-κB signaling and elevating oxidative stress and inflammation. The study also found that the joint administration of metronidazole and melatonin served as a promising therapeutic strategy for treating atherosclerotic cardiovascular diseases, particularly in the context of circadian disruption [83].

Additionally, a recent study delved into the role of *P. gingivalis* in smooth muscle cell (SMC) apoptosis and macrophage efferocytosis, which contribute to the acceleration of atherosclerosis. It was shown that *P. gingivalis* induces greater apoptosis in SMCs than in endothelial cells through activation of the TLR2 pathway. A notable finding was the extracellular release of miR-143/145 from *P. gingivalis*-infected SMCs, which were captured by macrophages. These miRNAs translocated into the macrophage nucleus and promoted Siglec-G transcription, inhibiting macrophage efferocytosis and impairing the clearance of apoptotic cells. The study also highlighted the development of a novel therapeutic approach using *P. gingivalis*-pretreated macrophage membranes to coat metronidazole and anti-Siglec-G antibodies, offering a potential dual treatment for both atherosclerosis and periodontitis [84]. Furthermore, a further study investigated the impact of *P. gingivalis* on the trimethylamine-N-oxide (TMAO) pathway, which is closely linked to cardiovascular diseases. The researchers used a mouse model to explore the effects of *P. gingivalis*, along with other oral pathogens like *Fusobacterium nucleatum* and *Streptococcus mutans*, on plasma TMAO levels and lipid metabolism. They found that infection with *P. gingivalis* significantly increased plasma TMAO levels and affected lipid metabolism, primarily by modulating the expression of hepatic flavin-containing monooxygenase 3 (FMO3). This led to elevated lipid factors such as triglycerides (TG), total cholesterol (TC), and non-esterified fatty acids (NEFA). The study confirmed that *P. gingivalis* played a more prominent role in elevating plasma TMAO levels compared to *F. nucleatum* and *S. mutans*, with profound effects on lipid metabolism in the liver. In vitro experiments with HepG2 cells revealed that *P. gingivalis*-LPS stimulation upregulated FMO3 expression, supporting the role of this bacterium in modulating lipid metabolism and increasing cardiovascular disease risk [85]. These findings reinforce the idea that oral pathogens not only contribute to local oral diseases but also play a significant role in systemic cardiovascular risk, highlighting the importance of addressing oral infections in the prevention and management of cardiovascular diseases.

#### 3.1.2. Thrombotic Risk

Oral pathogens can also elevate cardiovascular risk by promoting thrombogenesis. During bacteremia, *Streptococcus sanguis* can induce platelet aggregation, forming endocardial vegetations in cases of infective endocarditis. This platelet activation may extend to coronary thrombosis, heightening myocardial infarction (MI) risk, particularly under hyperlipidemic conditions, where thrombogenic responses are more pronounced [86].

Certain periodontal pathogens, including *P. gingivalis*, *Eikenella corrodens*, and *Prevotella intermedia*, can invade coronary endothelial and smooth muscle cells, exacerbating the inflammatory response tied to atherosclerosis. Once within coronary artery cells, these bacteria can interfere with cellular function and promote plaque development, establishing a direct connection between oral infections and cardiovascular complications [87]. Apical lesions of endodontic origin (ALEOs) have been correlated with elevated high-sensitivity C-reactive protein (hsCRP) levels, a marker of systemic inflammation and cardiovascular risk. Elevated interleukin-6 (IL-6) and matrix metalloproteinase-8 further substantiate the inflammatory burden of ALEOs, which disrupt endothelial function, promote adhesion molecule expression, and exacerbate cardiovascular risk [88].

Tooth loss due to chronic oral infections is linked to an increased risk of coronary heart disease (CHD), as the inflammatory burden from oral infections promotes endothelial dysfunction and encourages thrombosis. The systemic effects of periodontitis-related bacterial endotoxins exacerbate this risk by advancing atherosclerosis [89]. Furthermore, *Streptococcus mutans*, found in cardiovascular tissues, is implicated in systemic inflammation, as it can migrate from the oral cavity to cardiac tissue, where it triggers immune responses and pro-inflammatory cytokines, leading to endothelial dysfunction and exacerbating atherosclerosis [90].

In experimental models across species like dogs, mice, rabbits, and monkeys, molecular mimicry, whereby similarities between microbial and host proteins elicit cross-reactive immune responses, has been suggested as a key mechanism linking periodontitis to CVD through the induction of heat-shock proteins (HSPs). These proteins can accelerate atherosclerosis by triggering immune cascades that affect cardiovascular tissues [91,92,93]. In an additional study, *P. gingivalis* infection in obese mice led to increased endothelial injury and a higher prevalence of apoptotic cells in aortic tissues compared to non-infected controls. Immunohistochemical analysis found *P. gingivalis* within the smooth muscle of the aorta, with a greater presence in obese mice, emphasizing the complex interactions between periodontal disease and CVD, especially in the context of other risk factors [94].

Additionally, several studies indicate that treating periodontitis may help reduce other CVD risk factors, such as lowering C-reactive protein (CRP) levels and improving endothelial function, both clinically and through surrogate measures [95]. Intervention trials have also facilitated further insights into mechanisms linking periodontal disease and CVD by targeting potential pathways to observe their impact on cardiovascular outcomes. For example, antibiotic interventions aimed at microorganisms found in atherosclerotic plaques have clarified the limited role of direct infectious etiology in CVD. New pathways involving programmed inflammation resolution have shown promise for modifying inflammatory processes without directly targeting bacterial infection [96].

**Figure 2:**
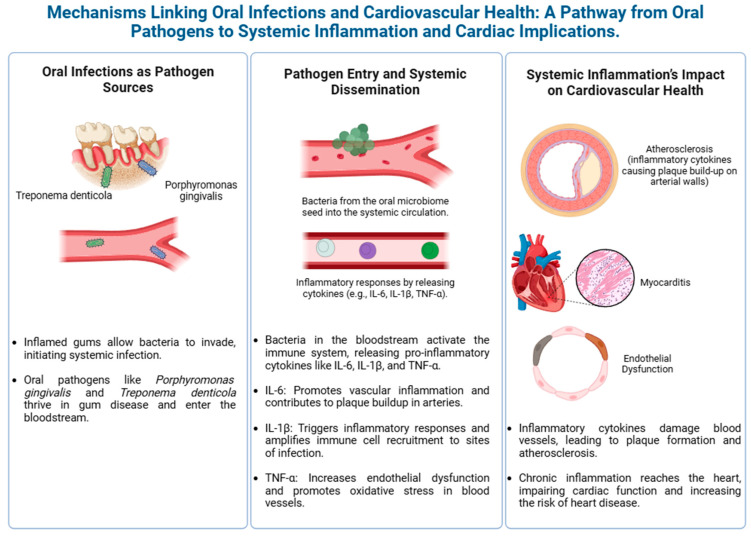
Mechanisms linking oral infections to cardiovascular disease.

Figure 2 highlights the potential link between oral health and cardiovascular health.

### 3.2. Experimental Evidence

One study exploring the relationship between dental extractions and bacterial endocarditis in rats found that periodontal disease substantially increases endocarditis risk. Periodontal disease was induced in rats using silk ligatures and a high-sucrose diet. Following this, sterile aortic valve vegetations were created, and 24 h later, the maxillary first molars were extracted. The results showed that 48% (14 of 29) of rats with periodontal disease developed bacterial endocarditis due to Streptococcus spp., while only 6% (1 of 15) of those with healthy periodontal conditions developed the condition. Notably, rats with periodontal disease that underwent catheterization without extraction showed no cases of endocarditis, emphasizing that dental extractions in periodontal disease increase the risk of systemic bacterial infection [97].

Additional studies in rats have reinforced the link between periodontitis and cardiovascular impairment, showing that ligature-induced periodontitis leads to alveolar bone loss, reduced ejection fraction, compromised myocardial relaxation, and increased heart rate. Histopathological findings of myocardial damage further highlight the interplay between chronic oral infections and cardiac health [10]. Another investigation demonstrated that melatonin treatment mitigated oxidative damage in cardiac tissue caused by periodontitis, reducing malondialdehyde (MDA) levels and increasing glutathione peroxidase (GSH-Px) activity. The melatonin-treated group also showed decreased bone loss, suggesting that melatonin may protect cardiac health in the context of oral infections [98].

The effects of apical periodontitis (AP) on cardiac function were examined in hypertensive rats, where AP exacerbated oxidative stress and cardiac impairment, marked by elevated superoxide anion levels and reduced superoxide dismutase activity. Hypertensive rats with AP also had larger AP areas, demonstrating the compounding impact of hypertension on cardiovascular complications in the presence of oral infections [77]. Another study in rats with experimentally induced periodontitis revealed that oxidative stress markers, such as malonylaldehyde and 8-hydroxy-2′-deoxyguanosine, were significantly elevated, indicating that periodontitis may drive cardiac remodeling and heart failure through increased reactive oxygen species production [99].

In Balb/c mice, periodontitis induced by molar ligation resulted in increased heart rates, arterial pressure variability, and decreased ejection fraction, indicating sympathetic overactivity and cardiac dysfunction. Elevated myocardial cytokines, including IL-17, IL-6, and IL-4, were also observed, correlating with findings in human studies linking periodontitis with cardiovascular risk [11]. In spontaneously hypertensive rats (SHR) and normotensive Wistar-Kyoto rats, periodontitis-induced alveolar bone loss was more severe in SHRs. Despite increased heart rate and arterial pressure in SHRs, periodontal disease unexpectedly lowered arterial pressure, likely due to increased plasma nitric oxide (NO) levels, demonstrating the complex relationship between periodontal disease and cardiovascular health [100].

Ligature-induced periodontitis in rats impaired endothelium-dependent vasodilation, a marker of endothelial dysfunction, and increased systemic inflammation, lipid profile deterioration, vascular superoxide production, and reduced nitric oxide synthase 3 (NOS-3) expression. These findings suggest a transient systemic and vascular inflammatory response following periodontitis, highlighting the model’s potential for studying the periodontitis–cardiovascular health connection [101].

The research using hyperlipidemic ApoE−/− mice demonstrated that oral infection with *Treponema denticola* accelerated atherosclerosis, increased aortic plaque area, reduced serum nitric oxide, and elevated oxidized LDL levels. This study underscores how oral infections may drive cardiovascular disease progression through changes in systemic inflammation and vascular health [81]. Similarly, chronic *Porphyromonas gingivalis* infection in ApoE-null mice resulted in alveolar bone loss, aortic inflammation, and elevated systemic markers, such as serum amyloid A and oxidized LDL, highlighting the inflammatory pathway by which periodontal disease may influence atherosclerosis [102].

Further research found that lipid A mutants of *P. gingivalis* allow the bacteria to evade TLR4-mediated immune responses, promoting vascular inflammation and macrophage accumulation, thereby accelerating atherosclerosis. This immune evasion strategy illustrates distinct mechanisms by which *P. gingivalis* contributes to both local bone loss and systemic inflammation [103]. The role of MCP-1, a pro-inflammatory chemokine, in atherosclerosis was explored in ApoE−/− mice lacking CCR2, which reduced lesion formation without affecting plasma lipids, linking MCP-1 to atherosclerosis development [104].

In models of juvenile periodontitis, elevated prostaglandin E2 (PGE2) and lipoxin A4 in crevicular fluids were observed, with neutrophils shown to produce lipoxins that reduce neutrophil recruitment and PGE2 levels in inflammation. These findings suggest that targeting neutrophil-mediated inflammation may limit periodontal pathogens’ impact on cardiovascular health [105]. Lastly, *P. gingivalis* infection has been shown to exacerbate inflammation and plaque formation in ApoE−/− mice, with MRI confirming elevated lipid and immune cell infiltration in infected mice. Immunization with heat-killed *P. gingivalis* prevented these changes, suggesting the potential for immunization as a preventive strategy [106]. The cardiovascular implications of oral infections in murine models, including mechanisms of inflammation, oxidative stress, and bacterial translocation, are summarized in Table 2.

### 3.3. Addressing the Gaps: Methodological and Translational Constraints of Murine Models

#### 3.3.1. Periodontal Ligature Model and Its Acute Nature

The periodontal ligature model effectively replicates the inflammatory and tissue-destructive processes observed in periodontitis, a dysbiotic oral disease linked to systemic inflammatory disorders. In this model, dysbiosis of the periodontal microbiota promotes an imbalance of microbial species, enabling pathogens like *Porphyromonas gingivalis* to evade immune responses and incite excessive inflammation. This cascade involves keystone pathogens subverting the host’s immune defenses with the help of accessory pathogens, eventually leading to overactivation by pathobionts and significant tissue destruction [12]. Key cytokines, such as interleukin-1 (IL-1) and tumor necrosis factor-alpha (TNF-α), play critical roles in amplifying the inflammatory response, driving bone resorption, and promoting connective tissue loss [107]. However, the acute inflammatory nature of this model limits its ability to fully emulate the chronic progression of human periodontitis. Despite these limitations, clinical and animal studies reveal significant systemic implications, with *P. gingivalis* and *Fusobacterium nucleatum* detected in extra-oral sites, such as aortic tissues and the placenta, where they are implicated in atherogenesis and adverse pregnancy outcomes [12]. By highlighting microbial immune evasion and cytokine-driven pathology, this model offers insights into periodontitis and its systemic impacts while underscoring the need for chronic models to better mimic human disease progression.

#### 3.3.2. Immune System Differences Between Rodents and Humans

While murine models are indispensable tools in immunological research, significant evolutionary differences between rodent and human immune systems challenge their translational reliability. Key discrepancies exist in both innate and adaptive immunity, including variations in leukocyte subsets, Toll-like receptors, cytokine profiles, and the signaling pathways of B and T cells. For instance, rodents rely on Ly49 inhibitory receptors in natural killer (NK) cells, while humans utilize killer immunoglobulin-like receptors (KIRs), reflecting divergent immune regulation strategies [108]. Similarly, chemokine receptor expression and cytokine signaling pathways, such as Th1/Th2 differentiation, show notable differences that can alter inflammatory responses. These distinctions affect the applicability of murine models in simulating complex human diseases, such as multiple sclerosis and delayed-type hypersensitivity, where multicomponent immune processes operate differently [108]. Moreover, murine responses to acute inflammatory stressors, such as infections or trauma, exhibit poor genomic correlation with human conditions, with studies revealing almost random alignment between murine and human orthologous genes [109]. This gap highlights the limitations of extrapolating findings from mice to humans in the context of inflammatory diseases. While murine models provide invaluable insights into fundamental mechanisms, researchers must account for these differences and complement them with human-based studies to ensure translational relevance.

#### 3.3.3. General Challenges of Translating Murine Findings to Humans

While rodents are invaluable for studying conserved biological processes and mechanisms, their translational applicability to human diseases is often limited by significant differences in genetics, physiology, and immune systems [110,111]. These differences influence how mice and humans respond to experimental interventions and disease processes. For example, mice primarily rely on tolerance mechanisms for pathogen defense, while humans exhibit stronger immune resistance strategies, leading to divergent outcomes in immune response studies [112]. Additionally, the networks linking genes to disease differ between species, complicating the extrapolation of murine findings to human pathologies. Outbred mouse stocks introduce further variability, as they lack consistent genetic quality control, which can lead to unexpected outcomes due to genetic mutations and mixed phenotypes within the same experimental group [113]. Although inbred strains provide genetic uniformity, they fail to capture the diversity seen in human populations. Recent advancements, such as humanized mouse models, aim to bridge these gaps by incorporating human immune system components, enhancing their relevance for studying human diseases [112]. However, careful consideration of these limitations is essential to optimize experimental designs and minimize resource wastage while ensuring reliable translational outcomes.

#### 3.3.4. Limitations of Murine Models in Atherosclerosis Research

Murine models are widely used in atherosclerosis research due to their ease of handling, rapid reproduction, and suitability for genetic manipulation. However, these models face significant translational challenges in replicating human cardiovascular diseases. Unlike humans, mice predominantly transport cholesterol in high-density lipoprotein (HDL) particles rather than low-density lipoprotein (LDL), which confers resistance to atherosclerosis [114]. Mice also lack cholesteryl ester transfer protein (CETP), a key factor in human lipoprotein metabolism, and produce unique bile acids that enhance cholesterol excretion, further protecting against atherogenesis [115]. Genetic and dietary modifications, such as high-fat diets and models like ApoE−/− and LDLr−/− mice, have been developed to overcome these limitations by mimicking human-like lipoprotein profiles [115]. Despite these advances, murine models fail to develop critical features of human atherosclerosis, such as coronary artery lesions, plaque rupture, and myocardial infarction, limiting their ability to simulate advanced disease stages [114,115]. While other species, such as rabbits, pigs, and non-human primates, offer closer anatomical and physiological similarities, they are costly, time-intensive, and pose ethical challenges [115].

#### 3.3.5. Future Research Directions

Future studies should develop chronic murine models to better simulate the progression of human diseases like periodontitis and its systemic effects. Advancements in humanized mouse models and organ-on-chip technologies can bridge species differences and enhance translational relevance. Multi-species comparative research and improved experimental designs will further clarify the mechanisms linking oral infections to cardiovascular diseases and strengthen clinical applications.

## 4. Conclusions

This narrative review highlights the essential role of murine models in understanding oral diseases and their systemic impacts, particularly on cardiovascular health. Models like ligature-induced periodontitis and peri-implantitis have clarified pathways linking oral inflammation to conditions such as endothelial dysfunction, oxidative stress, and atherogenesis. Oral pathogens, including *Porphyromonas gingivalis* and *Aggregatibacter actinomycetemcomitans*, play a significant role in driving systemic inflammation and cardiovascular dysfunction. While invaluable, murine models face translational limitations due to differences in immune responses and disease progression between rodents and humans. Acute models, like the periodontal ligature model, may not fully replicate chronic human diseases. Nonetheless, these models remain critical for uncovering mechanisms and testing therapies.

Future efforts should focus on developing chronic and humanized models, incorporating advanced imaging and multi-omics techniques to enhance translational relevance. Collaboration between basic and clinical researchers is crucial to bridge knowledge gaps and improve therapeutic strategies for oral and cardiovascular health. Addressing these challenges will ensure that murine models continue to advance understanding of the oral–systemic connection and contribute to innovative preventive and therapeutic approaches for global health.

## Figures and Tables

**Table 1:** Summary of experimental models of oral infections in rodents.

Model Type	Species	Procedure/Methodology	Duration	Key Findings	Reference
Periodontal Ligature Model	Wistar Rats, C57BL/6 Mice	Ligature placed around teeth (e.g., inferior frontal teeth in Wistar rats or left maxillary second molar in C57BL/6 mice) to induce gingival irritation, plaque accumulation, and periodontitis.	14 days (rats), 1–8 days (mice)	Induced significant gingival inflammation, plaque accumulation, alveolar bone loss, and periodontal disease.	[16,17]
Elastic Band Periodontal Model	Mice	An elastic band (0.3 mm) was inserted between the molars to induce mechanical stress and periodontal inflammation.	1 week on diet, followed by a period of elastic band application	Periodontal inflammation was induced depending on dietary salt intake (low, normal, or high salt).	[18]
Ligature and Oral Gavage Model	Mice	Ligature placed around molars and oral inoculation with *Porphyromonas gingivalis* or *Fusobacterium nucleatum*.	45–60 days	The ligature model induced significant inflammation and bone resorption, while gavage confirmed bacterial colonization with minimal tissue inflammation.	[19]
Bisphosphonate-Related Osteonecrosis of the Jaw (BRONJ) Model	Rats	Ligature placed around molars to induce periodontal disease, with concomitant zoledronic acid treatment to simulate BRONJ.	Not specified	Induced osteonecrosis in the jaw, confirmed by micro-CT and histology, mimicking human BRONJ pathology.	[20]
Zoledronate-Induced Osteonecrosis of the Jaw (ONJ) Model	C57BL/6J Mice	High-dose zoledronate (540 μg/kg) injection followed by molar extraction.	1 week post-injection, followed by wound monitoring	Delayed wound healing and hyperplasia in zoledronate-treated wild-type mice, while *γδ T* cell-deficient mice showed better healing despite ONJ lesions.	[21]
Orthodontic Tooth Movement (OTM) Model	Wistar Rats	A constant force (0.25 N) was applied to the upper molar using a NiTi coil spring, secured between the upper incisors for up to 4 weeks.	Up to 4 weeks	Induced significant tooth movement, with root resorption observed during extended durations.	[22]
Root Resorption Model	Wistar Rats	A NiTi closed-coil spring applied 50 g of force to the left molar to induce root resorption.	21 days	Induced root resorption, confirmed by histological analysis of tooth and periodontal tissue.	[39]
Periapical Lesion Induction Model	Sprague-Dawley Rats	Pulpal exposure of molars with a dental bur to induce infection and allow oral environmental exposure.	Variable	Induced formation of periapical lesions, mimicking natural periapical infection progression.	[24]
Mandibular Incisor Trimming Model	CD-1 Mice	Periodic trimming (~1 mm every other day) of mandibular incisors using orthodontic wire clippers.	2–6 weeks	Affected occlusion and jaw mechanics, with different outcomes based on diet (pellet vs. soft dough diet).	[29]
Unilateral Anterior Crossbite Model	C57BL/6J Mice	Metal tubes bonded onto left maxillary and mandibular incisors to induce unilateral anterior crossbite.	3 weeks	Induced significant tissue degradation in the temporomandibular joint (TMJ), varying by diet size (small particles vs. large pellets).	[25]
Forced Mouth Opening Model	C57BL/6 Mice	Stainless steel springs were used to force mouth opening (0.25–0.50 N force) for 5 consecutive days	5 days	Increased orofacial sensitivity, with higher forces causing significant cartilage degeneration typical of temporomandibular joint osteoarthritis (TMJ-OA).	[30]
Maxillary Implantation Model	Sprague-Dawley Rats, CD1 Mice	Extraction of maxillary molar followed by implantation of titanium screws or implants, with occlusal relief on opposing molars to prevent interference.	Variable	Assessed osseointegration and peri-implant healing, with studies focusing on factors such as bone formation, inflammation, and wound closure.	[31,32]
Peri-implantitis Model	C57BL/6J Mice	After implant placement and osseointegration, silk ligatures were placed around the implants to induce peri-implantitis.	12 weeks post-ligation	Induced significant peri-implant bone loss, mimicking clinical peri-implantitis.	[34]
Diabetic Peri-implantitis Model	Sprague-Dawley Rats	Type 2 diabetes was induced via a high-fat diet and streptozotocin injections, followed by peri-implantitis induced by LPS injections into the implant sulcus.	Variable	Significant inflammatory response and bone resorption observed, simulating peri-implantitis in diabetic conditions.	[35]
Oral Floor Tumor Model	Female BALB/c-NU Mice	Tumor cells (2 × 10^6^ or 8 × 10^5^) in Matrigel/HBSS were injected into the floor-of-mouth region under anesthesia. Once tumors reached 0.8 cm, surgical excision was performed with margins, confirmed via imaging.	Variable	Allowed for detailed investigation of oral floor tumor growth, excision, and recurrence, using imaging to confirm complete resection.	[36]
Simulated Alveolar Cleft Model	Rats	A 3.3 mm bone defect was surgically created in the anterior maxilla to simulate an alveolar cleft. Defects were treated with no graft, bHA, bHA with undifferentiated MSCs, or bHA with osteogenically differentiated MSCs.	Variable	The model provided insights into bone regeneration, showing enhanced bone formation in defects treated with osteogenically differentiated MSCs combined with bone graft material.	[37]
Tooth Extraction Model	Mice	The upper right incisor was extracted under anesthesia using a dental probe and tweezers. Post-extraction, the maxillae were collected at 0, 7, 14, and 21 days for micro-CT, histological, and molecular analyses.	0 h, 7, 14, 21 days	Allowed detailed investigation of post-extraction healing and bone remodeling using advanced imaging and molecular analyses.	[40]
PCL Biomembrane Implantation in Maxillary Defects Models	Mice	Maxillary bone lesions were created in the diastemal area, followed by the implantation of functionalized BMP-2/Ibuprofen scaffolds. Controls with no scaffolds were also included. The mucosa was closed using biological glue.	Not specified	Provided a platform for evaluating the effects of functionalized biomaterials on bone healing and regeneration.	[41]
Oral Mucosa Wound Healing Model	Sprague-Dawley Rats	Bilateral incisions were made in the anterior edentulous maxilla. Enamel Matrix Protein Derivative (EMD) was injected into the soft tissue on one side, while the contralateral side served as a control.	5–9 days	EMD treatment enhanced vascularization, collagen synthesis, and expression of growth factors and cytokines, improving oral mucosal wound healing.	[42]
FRICTION Model	BALBc and C57Bl/6 Mice	A chromic gut suture was inserted orally to irritate the V2 branch of the trigeminal nerve, modeling chronic neuropathic pain without visible signs of injury. Mechanical hypersensitivity and behavioral changes were monitored over 100 days.	Up to 100 days	Induced long-term mechanical hypersensitivity and behavioral changes, serving as a robust model for preclinical testing of non-opioid pain therapies.	[43]
Tunable Mechanical Overload Model	Holtzman Rats	Jaw-opening forces (2 N and 3.5 N) were applied daily for 1 h over 7 days, followed by a rest period. Orofacial sensitivity and TMJ degeneration were assessed.	7 days loading, assessed at 14 days	3.5 N loading induced persistent pain and TMJ cartilage degeneration, upregulating inflammatory markers, while 2 N loading did not induce chronic pain or significant inflammation.	[44]
4NQO-Induced Oral Mucosal Carcinogenesis Models	Sprague-Dawley Rats, C57BL/6JNarl Mice	4-Nitroquinoline-1-oxide (4NQO) was applied to the palatal and tongue mucosa in rats or dissolved in the drinking water for mice. Mice were also treated with arecoline hydrobromide.	Variable	Induced oral mucosal carcinogenesis and neoplastic changes in tongue and palatal tissues.	[45,46]
Osteonecrosis of the Jaw (ONJ) Model	Mice	High doses of zoledronic acid were administered, followed by pulpal exposure of mandibular molars to create periapical disease.	8 weeks	Induced ONJ in mandibles, with radiographic and histological analyses showing bisphosphonate effects on bone.	[48]
Bisphosphonate-Related and Denosumab-Related Osteonecrosis of the Jaw (BRONJ and DRONJ) Models	C57BL/6 Mice	Zoledronic acid (BRONJ) or anti-RANKL monoclonal antibody (DRONJ) injections were administered biweekly, followed by molar extraction.	4 weeks	Both models induced osteonecrosis in maxillae, with bone loss observed post-extraction and treatment.	[49]
Oral Ulcers Model	Wistar Rats	Ulcers were induced using 100% acetic acid applied to the gingival or lingual mucosa following sialoadenectomy. Another model involved the application of formocresol to the gingiva in rats.	Variable	Ulcer formation and healing patterns were analyzed after various treatments (e.g., ghrelin, saline).	[50,51]
Inflammatory Bone Loss Model	Mkp-1+/+ and Mkp-1−/− Mice	LPS from *Aggregatibacter actinomycetemcomitans* was micro-injected into gingiva three times weekly to induce alveolar bone loss.	4 weeks	Induced inflammatory bone loss, providing insights into the role of Mkp-1 in bone remodeling and inflammation.	[65]
Peri-implant Mucositis and Peri-implantitis Model	C57BL/6J Mice	Mice received custom titanium implants post-molar extraction. *P. gingivalis* LPS injected into peri-implant mucosa to induce mucositis and peri-implantitis.	6 weeks	Induced peri-implant mucositis and bone loss, replicating peri-implant diseases seen in clinical settings.	[61]
Dentin Protein Immunization Model	NIH Swiss Female Mice	Mice were immunized intraperitoneally with mouse dentin protein emulsified in Complete Freund’s Adjuvant, followed by weekly boosters with dentin from Sprague-Dawley rats.	4–10 weeks	Induced antibody production against dentin, modeling immunological responses to dental proteins.	[52]
Induced Buccal Mucosa Lesion Model	BALB/c Mice	Areca nut extract was applied to the buccal mucosa twice daily for 300–600 days to induce lesions. Control mice received saline.	300–600 days	Induced buccal mucosa lesions, providing insights into the effects of areca nut extract on oral tissue over long-term exposure.	[53]
Deproteinized Bovine Bone and Bioactive Glass-Induced Guided Tissue Regeneration (GTR) Model	Rats	Hemispherical Teflon capsules packed with either deproteinized bovine bone (Bio-Oss^®^) or bioactive glass (Biogran^®^) were placed on the mandibular ramus to evaluate long-term bone regeneration.	1 year	Limited bone formation was observed with grafting materials, while controls showed substantial new bone formation, highlighting the inhibitory effects of the grafts.	[54]
Oral Submucous Fibrosis (OSF) Model	Rats	Daily injections of bleomycin into buccal mucosa induce fibrosis, with histopathological features assessed for collagen deposition and ultrastructural changes.	2–8 weeks	Induced oral submucous fibrosis, mirroring the fibrotic changes observed in human OSF, validating the model for further research.	[55]
Bacterial Peri-Implantitis Model	Sprague-Dawley Rats	Titanium implants were placed in the maxilla, followed by bacterial lavage with *Streptococcus oralis* and *Aggregatibacter actinomycetemcomitans* to induce peri-implantitis.	3 months	Significant bone loss and peri-implant inflammation, replicating human peri-implantitis.	[67]
Dental Implant Biofilm Formation Model	Sprague-Dawley Rats	Custom-made dental implants were placed, followed by inoculation with human-derived biofilm-forming bacteria: *Streptococcus oralis*, *Fusobacterium nucleatum*, and *Porphyromonas gingivalis*.	6–7 weeks	Reliable biofilm quantification and assessment of peri-implant mucositis.	[68]
Induced Oral Cancer Model	Mice	Mice with inducible K-rasG12D mutations in stratified epithelia were crossed with K14.CrePR1 or K5.CrePR1* mice. RU486 treatment activated K-ras in oral tissues, causing squamous papillomas.	Not specified	Developed benign squamous papillomas, mimicking early stages of human oral tumorigenesis.	[56]
Induced Cyclin D1/p53 Tumorigenesis Model	Mice	Transgenic mice expressing human cyclin D1 were generated and backcrossed with p53 heterozygous mice. A subgroup was treated with sulindac to assess its chemopreventive potential.	3 months	Enhanced tumorigenesis with combinations of cyclin D1 and p53 expression. Sulindac treatment showed chemopreventive effects.	[57]
TGF-β3 Knockout Model for Palatal Fusion Studies	Mice	Knockout (TGF-β3−/−), heterozygous (TGF-β3+/−), and wild-type (TGF-β3+/+) embryos were generated to study the role of TGF-β isoforms in palatal fusion using in vitro organ culture methods.	Embryonic days 12.5–16.5	Provided insights into the roles of TGF-β isoforms in palatal fusion, with different growth factors influencing palatal shelf development and fusion.	[58]
Infection-Induced Periapical Lesion Model	IL-4−/− and IL-10−/− Mice	Exposed molars infected with a mix of endodontic pathogens (*Prevotella intermedia*, *Fusobacterium nucleatum*, *Peptostreptococcus micros*, and *Streptococcus intermedius*).	21 days	Significant bone resorption and inflammation, modeling periapical periodontitis in genetically engineered mice.	[59]
Polymicrobial Periodontal Infection Model	Rats	Oral inoculation with a polymicrobial mixture of *Porphyromonas gingivalis*, *Treponema denticola*, *Tannerella forsythia*, and *Fusobacterium nucleatum* to simulate chronic periodontal disease.	Variable	Increased alveolar bone loss and systemic immune response, replicating polymicrobial human periodontal disease.	[60]
Pathogen-Induced Periodontal Disease Model	Mice	A ligature was placed around the first maxillary molars, followed by oral inoculation with *Porphyromonas gingivalis* and *Fusobacterium nucleatum*	1–4 weeks	Induced periodontal inflammation and bone loss, resembling human periodontal disease.	[63]
*Candida albicans* Colonization Model	Rats	Inoculation with *Candida albicans* on the tongue followed by metronidazole treatment.	7, 15, and 30 days post-inoculation	Persistent colonization of *C. albicans*, modeling oral candidiasis.	[62]

**Table 2:** Cardiovascular implications of oral infections in murine models.

Mechanism	Model and Pathogen	Cardiovascular Findings	Reference
Systemic Inflammation and Cytokine Elevation	Ligature-induced periodontitis (*Porphyromonas gingivalis*)	Increased levels of pro-inflammatory cytokines (IL-6, TNF-α) were linked to endothelial dysfunction and vascular inflammation.	[14,69]
	*Aggregatibacter actinomycetemcomitans* infection	Elevated IL-6, IL-8, TNF-α; systemic inflammation; increased adhesion molecules ICAM-1, E-selectin in aortic tissue.	[71]
Direct Bacterial Translocation to Cardiovascular Tissues	Periodontitis with *P. gingivalis* inoculation	Detection of *P. gingivalis* DNA in aortic tissue; promotes vascular inflammation and contributes to atherosclerosis.	[70,73]
	*Treponema denticola* infection	Elevated IgG antibodies; increased vascular inflammation; higher oxidized LDL and reduced nitric oxide levels, promoting atherosclerosis.	[81]
Oxidative Stress and Endothelial Dysfunction	Apical periodontitis in hypertensive rats	Elevated superoxide anion levels and decreased antioxidant enzyme activity led to myocardial damage and impaired cardiac dynamics.	[77,78]
	Ligature-induced periodontitis in ApoE−/− mice	Increased oxidative stress markers (malondialdehyde, 8-hydroxy-2′-deoxyguanosine) contribute to cardiac remodeling.	[99,101]
Thrombogenesis and Platelet Aggregation	*Streptococcus sanguis* bacteremia	Platelet aggregation linked to endocardial vegetation formation; potential for coronary thrombosis under hyperlipidemic conditions.	[86]
Direct Cardiac Impact and Myocardial Inflammation	*P. gingivalis* bacteremia	Induces myocarditis and myocardial infarction; elevated levels of IL-1β, IL-6, IL-17A, and TNF-α in heart tissue, with neutrophil infiltration.	[80]
Periodontitis and Blood Pressure	Spontaneously hypertensive rats with periodontitis	Lowered arterial pressure was observed, possibly due to increased nitric oxide levels despite increased systemic inflammation.	[100]
Cardiac Remodeling due to Oral Infection	Ligature-induced periodontitis in Balb/c mice	Increased heart rate and arterial pressure variability; myocardial cytokines (IL-6, IL-4, IL-17) elevated, correlating with cardiac dysfunction.	[11]

## Data Availability

The collected literature is available on request.
